# Local atrial bipolar electrogram voltage drops during cardiac magnetic resonance guided catheter ablation of typical atrial flutter: Associations with delivered radiofrequency energy and peri-procedural imaging

**DOI:** 10.1016/j.hroo.2024.08.015

**Published:** 2024-09-06

**Authors:** Hedwig M.J.M. Nies, Dominik Linz, Geertruida P. Bijvoet, Robert J. Holtackers, Justin G.L.M. Luermans, Kim E.H.M. van der Velden, Joachim E. Wildberger, Kevin Vernooy, Sander M.J. van Kuijk, Casper Mihl, Sevasti-Maria Chaldoupi

**Affiliations:** 1 Cardiovascular Research Institute Maastricht (CARIM), Maastricht University, Maastricht, The Netherlands; 2 Department of Radiology & Nuclear Medicine, Maastricht University Medical Centre, Maastricht, The Netherlands; 3 Department of Cardiology, Maastricht University Medical Centre, Maastricht, The Netherlands; 4 Faculty of Health and Medical Sciences, Department of Biomedical Sciences, University of Copenhagen, Copenhagen, Denmark; 5 Department of Anesthesiology, Maastricht University Medical Centre, Maastricht, The Netherlands; 6 Department of Clinical Epidemiology and Medical Technology Assessment, Maastricht University Medical Center, Maastricht, The Netherlands

**Keywords:** Ablation lesion assessment, Atrial flutter, Durable ablation lesion formation, Interventional cardiac MRI (iCMR), Late electrical reconnection, Local atrial bipolar electrogram, Radiofrequency catheter ablation

## Abstract

**Background:**

Cardiac magnetic resonance (CMR)-guided catheter ablation of the cavotricuspid isthmus (CTI) has been proven feasible, but determinants of local electrogram (EGM) voltage drops during radiofrequency (RF) applications are unknown.

**Objective:**

The purpose of this study was to investigate local atrial bipolar EGM voltage drops and the association with delivered RF energy and anatomical information derived from peri-procedural CMR imaging.

**Methods:**

In consecutive patients undergoing CMR-guided CTI ablation procedures, relative EGM voltage drops for RF applications ≥20 seconds were calculated. Pre- and post-ablation CMR imaging was performed. Associations of relative EGM voltage drops with patient characteristics, delivered RF energy, and CTI anatomy were analyzed.

**Results:**

In total, 216 RF applications were evaluated from 12 patients (18 ± 5 applications/patient). EGM voltage amplitude at baseline was significantly higher in the group with the strongest relative EGM voltage drop (*P* < .05), whereas RF ablation settings (duration, power, temperature) and lesion characteristics (impedance drop, slope of impedance drop) did not differ. The EGM voltage amplitude at baseline (*P* < .001), left ventricular ejection fraction (LVEF) (*P* = .020), right atrium volume index (RAVI) (*P* = .027), and CTI line length (*P* = .026) showed the strongest association with relative EGM voltage drop. Four of 12 patients (33%) underwent a re-do procedure, 2 patients showed a regional late reconnection, which could be visually identified in the T_2_-weighted images (T2WI) of the index procedure.

**Conclusion:**

Local EGM voltage amplitude, LVEF, RAVI, and CTI length are associated with relative EGM voltage drop during CMR-guided CTI ablation. Post-ablation CMR imaging during the index procedure may help to identify areas of late reconnection.

## Graphical abstract

**Figure:**
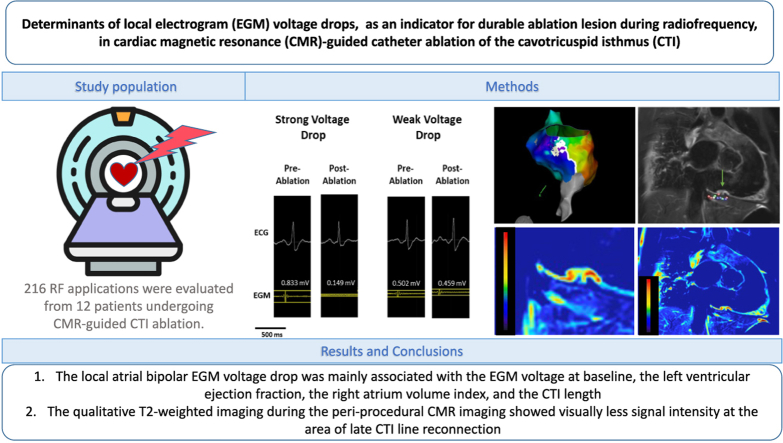

**Key Findings**

▪ During cardiac magnetic resonance (CMR)-guided catheter ablation of the cavotricuspid isthmus (CTI), the local atrial bipolar electrogram (EGM) voltage drop, as an indicator for durable ablation lesion, is associated with the EGM voltage at the baseline, left ventricular ejection fraction, right atrium volume index, and CTI length.
▪ The length of the ablation CTI line is shorter compared with the anatomically CMR-determined CTI length, indicating that some parts of the anatomical CTI do not have any atrial electrical signals and do not require ablation to achieve complete electrical block along the CTI.
▪ The location of electrical gaps during the re-do procedures attributable to late reconnection of the CTI line overlapped areas with less signal intensity in the visual assessment of the qualitative T2-weighted image derived from the index CMR-guided catheter ablation procedure.

## Introduction

Radiofrequency (RF) catheter ablation of the cavotricuspid isthmus (CTI) is the cornerstone of the typical atrial flutter (AFL) treatment.^1^ An ablation line from the tricuspid valve (TV) annulus toward the vena cava inferior (VCI) with transmural lesions without damage of the adjunct tissue is required to achieve successful and safe ablation outcomes. During conventional CTI catheter ablation procedures, an electroanatomic mapping (EAM) system provides real-time spatiotemporal information on the catheter tip location, contact tissue force, and force direction to ensure an accurate and stable positioning of a contact-force sensing catheter.^2^ Additionally, to guide RF energy delivery, an “ablation index” incorporating power, ablation duration, impedance drop, and catheter stability has been developed and shown to improve lesion size and ablation efficacy.^3^^,^^4^

Recently, cardiac magnetic resonance (CMR)-guided CTI catheter ablation has been proven feasible in the treatment of typical AFL, and it enables real-time anatomy and tissue visualization, which may have the potential to identify locations of late CTI electrical reconnection.^5^, ^6^, ^7^ Although currently available CMR-compatible catheters with 2 integrated magnetic resonance receiver coils can be visualized and tracked real-time during a CMR-guided ablation procedure through a dedicated imaging guidance software,^8^^,^^9^ contact tissue force information is not provided. Additionally, no “ablation index” has been developed as a more robust predictive marker of durable ablation lesion formation. Therefore, electrophysiologists operating this catheter ablation procedure currently rely exclusively on tactile feedback of the catheter, temperature of the catheter tip during RF application, and established electrophysiological parameters such as local bipolar electrogram (EGM) voltage amplitudes, and the response to ablation. However, the association of EGM voltage drops, as an indicator of durable lesions, with periprocedural RF ablation settings, CMR imaging of the target CTI anatomy, and CMR imaging of the acute ablation lesions have not been studied yet in the interventional CMR (iCMR) suite environment.

In this study, we analyzed local atrial bipolar EGM voltage drops as an indicator for durable ablation lesion during CMR-guided catheter CTI ablation in patients treated for typical AFL and studied their association with delivered RF energy and periprocedural CMR imaging–derived information during the index procedure. Additionally, locations of late electrical CTI reconnection sites during clinical re-do procedures were compared with electrophysiological and CMR imaging data derived from the index procedure.

## Materials and methods

Twelve consecutive patients scheduled for CMR-guided CTI ablation to be treated for symptomatic typical counterclockwise AFL were prospectively included between February 2021 and September 2022. Exclusion criteria were age <18 years, the general CMR contraindications (eg, metallic implant and body weight >130 kg), known severe allergy to gadolinium contrast agents, and renal failure with an estimated glomerular filtration rate ≤30 mL/min/1.73 m^2^. Transthoracic echocardiography was performed for all patients within 3 years before the procedure. This study adhered to the declaration of Helsinki and was approved by the local ethical committee (NL74812.068.20). All patients provided written informed consent.

### Periprocedural CMR-guided catheter ablation workflow

All CMR-guided catheter ablation procedures were performed in a preexisting CMR environment that was transformed into an iCMR suite and followed the procedural workflow as described earlier.^10^^,^^11^ Briefly, after general anesthesia with intubation and obtaining vascular femoral access with 2 sheaths (10 F and 12 F) outside the magnetic resonance imaging (MRI) scanner room, the patient was transferred into a 1.5 T clinical MRI system (Ingenia; Philips Healthcare, Best, The Netherlands). Two CMR-compatible, bipole irrigated-tip catheters with integrated magnetic resonance receiver micro coils for active catheter tracking (Vision-MR Ablation Catheter, Imricor Medical Systems, Inc., Burnsville, MN) were used and connected to an Advantage-MR EP Recorder/Stimulator System (Imricor Medical Systems, Inc., Burnsville, MN), all approved for clinical use. The catheters were introduced via the femoral sheaths, and their visualization and navigation took place using an CMR-specific research EAM/navigation system (iSuite; Philips Healthcare, Best The Netherlands) that enables active catheter tracking and integration of CMR imaging and electrophysiological information throughout the procedure. Finally, the iSuite system allows the operator to annotate the ablation points and project those on the CMR images, creating an ablation point line. The diagnostic catheter was placed in the coronary sinus (CS); the second catheter was used as ablation catheter.

#### Electrophysiological study and radiofrequency ablation

A design line was created to identify the optimal ablation path according to our institutional strategy, which has been described previously.^11^ After positioning of the catheter tip on the target location, the catheter position was annotated on the iSuite system. During RF energy delivery, no real-time active catheter tracking was used to prevent noise on the EGMs. Catheter stability was judged based on EGM interpretation by the operating electrophysiologist during RF energy delivery and reconfirmed by real-time active tracking, immediately before and directly after RF ablation. According to the manufacturer’s recommendations, RF ablation was performed by delivering 30–50 W titrated manually to achieve a tip temperature of 35°C–0°C for at least 20 seconds and not exceeding 120 seconds (irrigation 17 mL/min).^12^ The RF delivery was immediately terminated in the event of catheter displacement, sudden impedance rise, or a catheter tip temperature of >40°C. Bidirectional CTI conduction block was confirmed by differential pacing, by double potentials >100 ms along the line, or when a color-coded activation map, performed manually with the iSuite system, showed a counterclockwise activation pattern around the TV annulus during pacing from the proximal CS catheter. After a waiting period of 30 minutes, in which the post-ablation CMR imaging was performed, the electrophysiologist (S. M. C. and D. L., 7 years’ clinical experience each) evaluated the durability of the CTI ablation. In case of electrical reconnection along the line, additional RF applications were applied at the site(s) where the electrical reconnection was judged to be by the operator based on the electrophysiological (EP) findings alone, while being blinded to the results of post-ablation CMR imaging, to achieve conduction block again.

#### CMR imaging

Baseline CMR imaging has been described previously.^13^ Details about CMR acquisition parameters are summarized in Supplemental Table 1.

In brief, an electrocardiogram (ECG)-triggered navigator-gated 3D whole-heart balanced steady-state free precession sequence is performed, which served as a roadmap for the iSuite EAM system. The CTI anatomy was visualized in the left anterior oblique, the right anterior oblique (RAO), and the transversal plane.^14^ A stack of 3 consecutive slices of the CTI line were predefined in all 3 imaging planes for the pre- and post-ablation imaging. Pre-ablation imaging included cine imaging, black-blood T_2_-weighted spectral presaturation with inversion recovery imaging, and T_1_ mapping sequences. After the initially CTI conduction block and during the 30-minute waiting period, post-ablation imaging of the acute ablation lesions was performed to visualize the tissue characteristics or changes of the ablated myocardium. First, T_2_-weighted imaging (T2WI) and T_1_ mapping sequences were acquired in exactly the same slice orientation as before ablation imaging. For optimal image quality, the T2WI, T1 mapping, and cine images were performed during end-expiratory ventilator stops (of the intubated patient under general anesthesia).

First-pass perfusion imaging was performed during administration (0.2 mmol/kg) of Gadobutrol (Gadovist; Bayer Pharmaceuticals, Berlin, Germany) to evaluate myocardial blood flow and perfusion. Approximately 10 minutes after contrast administration, 2D or 3D dark-blood late gadolinium enhancement (LGE) images were obtained using a standard ECG-triggered breath-holding phase-sensitive inversion-recovery sequence.^15^^,^^16^ All LGE images were acquired in mid-diastole and end-expiratory ventilator stops or during breath apnea after pre-oxygenation as the patients were intubated. Finally, direct post-ablation imaging enables detection of possible early complications (eg, increase in pericardial fluid compared with pre-procedural imaging).

All CMR images were exported after completion of the ablation procedure and analyzed retrospectively using Sectra Workstation IDS7 (version 24.2, Sectra AB, Linköping, Sweden). The CTI length was measured based on anatomical landmarks during ventricular diastole and systole using cine RAO images. Post-ablation T_2_-weighted and dark-blood LGE CMR images were visually assessed for the presence of edema and LGE, respectively. In addition, the post-ablation T_2_-weighted images were postprocessed into color-coded images to highlight the signal intensity differences. All CMR images were analyzed blinded to EP parameters and iSuite information.

## Discharge, follow-up, and re-do procedures

The patients were discharged from the hospital the day after the procedure if no complications arose. Follow-up visits after 3, 6, and 12 months were scheduled at the outpatient clinic, where an ECG was routinely performed and Holter monitoring was used if indicated. With symptomatic recurrence of AFL or other symptomatic atrial arrhythmias, a re-do procedure in the conventional EP laboratory was performed. A high-density EAM of the right atrium with the use of the PentaRay® high-density mapping catheter on CARTO system (Biosense Webster, Inc., Diamond Bar, CA) during CS pacing was performed to evaluate the CTI ablation line. In case of reconnection, segmental RF ablation would be applied to achieve bidirectional electrical blockage along the CTI line.

### Data analysis

**Figure 1:**
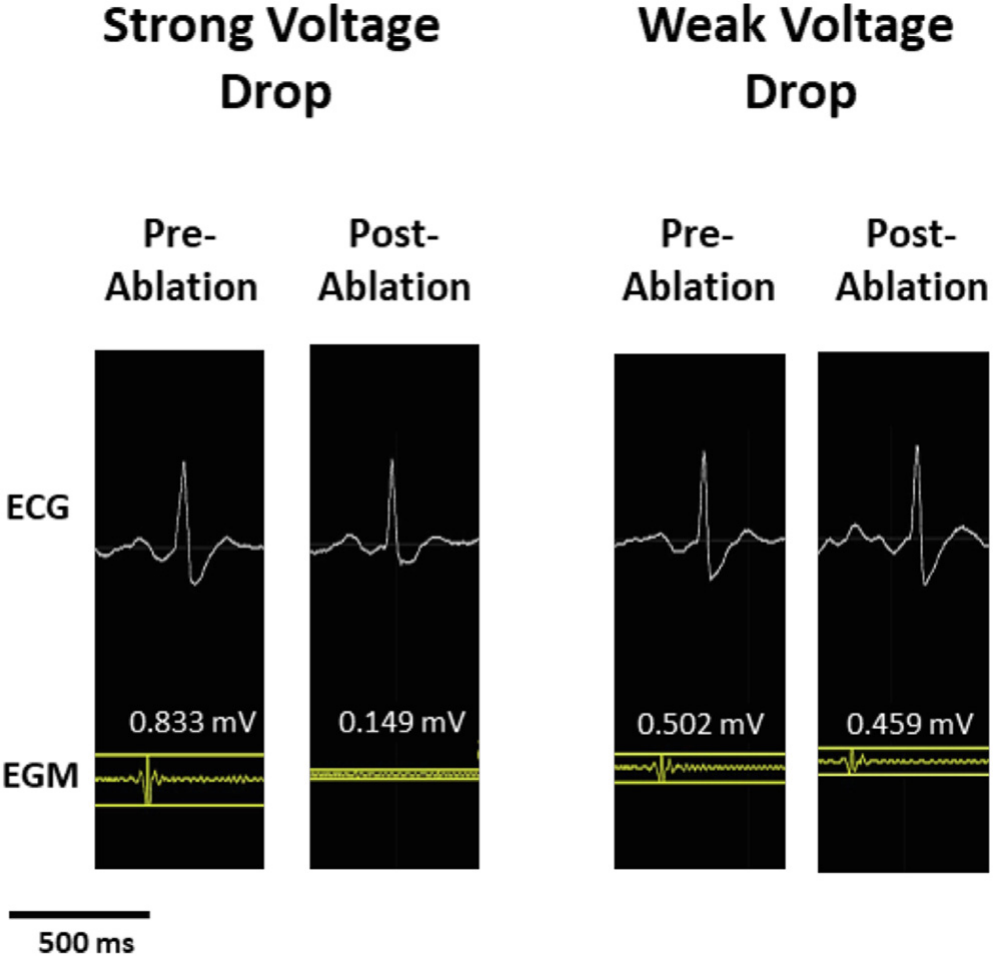
Representative examples of intracardial EGM before and after ablation with strong voltage drop (left panel) and weak voltage drop (right panel) pre- and post- ablation. ECG = electrocardiogram; EGM = electrogram.

Of all CMR-guided CTI ablation procedures, the EGM voltage amplitude was measured at the start and end of each RF application, with an RF energy delivery ≥ 20 seconds^17^ by experienced electrophysiologists (Figure 1). The absolute [(start EGM voltage amplitude) – (end EGM voltage amplitude)] and relative [(absolute voltage amplitude drop)/(start EGM voltage amplitude)] EGM voltage drop was calculated. RF applications were only included for further analyses when the relative EGM voltage drop was ≥0%.

**Figure 2:**
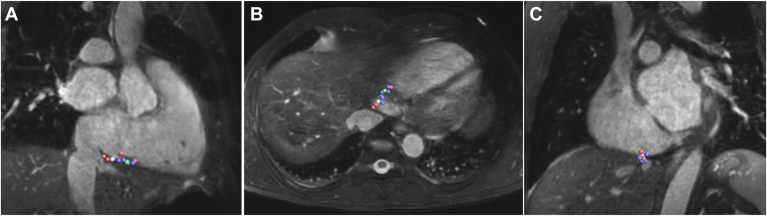
Color-coded distribution of RF ablation lesions according to the relative EGM voltage drop within 1 representative patient (white representing the weakest, green the intermediate weak, purple the intermediate strong, and red the strongest drops). Location of all RF applications are projected on baseline **A:** RAO, **B:** TRA, and **C:** LAO CMR images using the iSuite system and are color-coded according to the respective relative EGM voltage drop quartiles. CMR = cardiac magnetic resonance; EGM = electrogram; LAO = left anterior oblique plane; RAO = right anterior oblique; RF = radiofrequency; TRA = transversal.

Within each patient, the RF applications were ranked based on the relative EGM voltage drop divided into quartiles. All RF applications were relocalized and projected on the CTI of the CMR image, using the iSuite system/software, and color-coded according to the respective relative EGM voltage drop, white representing all points in the first quartile (“weakest”), green in the second quartile (“intermediate weak”), purple in the third quartile (“intermediate strong”), and red in the fourth quartile (“strongest”) (Figure 2). The CTI was divided into 3 equally large anatomical sections: (A) TV annulus section, (B) mid-section, and (C) VCI section. Additionally, relative EGM voltage drops (according to quartiles and used as a continuous variable) were related to mean RF power, mean duration, mean temperature, absolute impedance drop, the regression coefficient (or slope) of impedance courses, CTI length, CTI thickness, and patient characteristics.

In patients with recurrent supraventricular arrhythmias that underwent a re-do conventional ablation procedure, the high-density EAM that was acquired during the re-do procedure was compared with the index CMR-guided catheter ablation procedure to retrospectively identify the areas with electrical reconnection along the CTI ablation line.

### Statistical analysis

Patient characteristics at baseline were summarized as mean and standard deviation for continuous variables, and as count and percentage for categorical variables. Characteristics of the CTI were compared within participants using the paired-samples *t* test.

To assess interobserver variability in a subset of 2 patients [34/216 (16%) ablation lesions] intracardiac bipolar electrograms were measured by 2 independent electrophysiologists (S. M. C. and D. L.). Interobserver agreement regarding endocardial bipolar voltage measurements was assessed by using 2-way random-effect model intraclass correlation coefficient (ICC) and Cohen’s kappa statistics.

Using univariable linear mixed-effects regression analyses, we estimated the association between RF characteristics and effective ablation lesion formation expressed as the relative voltage reduction. We used a random intercept for patient number to account for the clustering of multiple measurements within patients and an autoregressive model of the first order to model spatial dependencies. All statistical analyses were performed using SPSS (version 27, IBM, Armonk, NY), with statistical significance set at *P* < .05.

## Results

### Patient characteristics and CTI

The patient characteristics of 12 included patients (62 ± 9 years, 83% male) are summarized in Table 1.

**Table 1:** Baseline patient characteristics

Patient characteristics	N = 12
Age (y)	62 ± 9
Male sex [n (%)]	10 (83)
BMI	26.3 ± 3.4
**Echocardiography**	
LVEF	
≥55% [n (%)]	10 (84)
45%–55% [n (%)]	1 (8)
≤45% [n (%)]	1 (8)
LAVI (mL/m^2^)	36 ± 9
RAVI (mL/m^2^)	32 ± 11

BMI = body mass index; LVEF = left ventricular ejection fraction; LAVI = left atrial volume index; RAVI = right atrium volume index.

The mean anatomical CTI length during ventricular diastole was significantly shorter than the mean anatomical CTI length during ventricular systole, 22.9 ± 4.5 mm vs 35.8 ± 3.9 mm (*P* < .001) when measured on CMR images. The mean thickness of the CTI was 3.3 ± 1.1 mm. Individual data are shown in Table 2.

**Table 2:** Individual anatomical characteristics of the CTI

Study number	Anatomical CTI length on MRI; systole (mm)	Anatomical CTI length on MRI; diastole (mm)	Anatomical CTI thickness on MRI (mm)	Length of ablation line on iSuite; TRA (mm)	Length of ablation line on iSuite; RAO (mm)
1	35	22	4	38	39
2	39	23	2	35	34
3	32	17	3	30	28
4	33	20	5	29	28
5	36	26	4	30	30
6	31	21	3	32	31
7	32	19	2	30	35
8	41	32	3	33	31
9	41	25	2	28	27
10	40	25	5	29	28
11	38	28	3	31	31
12	31	17	4	28	27
**Mean**	**35.8 ± 3.9**	**22.9 ± 4.5**	**3.3 ± 1.1**	**30.9 ± 3.6**	**31.1 ± 3.1**

CTI = cavotricuspid isthmus; MRI = magnetic resonance imaging; RAO = right anterior oblique plane; TRA = transversal plane.

The length of the ablation line along the CTI measured on iSuite was consistent between the transversal and the RAO planes (30.9 ± 3.6 mm and 31.1 ± 3.1 mm, respectively; *P* = .637), significantly shorter than the anatomical CTI length measured on CMR image during systole, and significantly longer than the anatomical CTI length measured during diastole (*P* = .006 and *P* < .001, respectively).

### Relative EGM voltage drops and associations with RF application characteristics

In total, 37 RF applications of the 253 RF applications from 12 patients were excluded because the relative EGM voltage drop was <0 %. This is a dropout of 15%. Therefore, 216 RF applications (18 ± 5 RF ablations per patient) were evaluated. The mean relative EGM voltage drop was 46.0% ± 24.0 %. The interobserver agreement regarding endocardial bipolar voltage measurements was good (intraclass correlation coefficient = 0.827; 95% CI, 0.570–0.923).

An overview of all RF application characteristics including duration, temperature, power, absolute impedance drop, and slope of impedance course of all RF applications divided according to relative EGM voltage drop quartiles is presented in Table 3. RF ablation settings and lesion characteristics were not significantly different between the relative EGM voltage drop quartiles (all comparisons nonsignificant). Only the EGM at baseline was significantly different between the quartiles (*P* = .001). The location of 208 RF applications could be localized on the CTI line (8 ablation lesions were not annotated during the procedure and could not be identified). No significant difference was found between the 4 EGM voltage drop quartiles regarding the spatial distribution pattern of RF applications on the 3 (TV annulus section, mid-section, and VCI section) CTI anatomical sections (*P* = .799).

**Table 3:** Relative EGM voltage drops (divided into quartiles; Q) and the association with RF ablation settings, lesion characteristics, and RF ablation location

EGM voltage drops and the association with RF ablation settings and lesion characteristics									
	Baseline EGM voltage (mV), mean ± SD	End of ablation EGM voltage (mV), mean ± SD	Absolute EGM voltage reduction (mV)	Relative EGM voltage reduction (%)	RF delivery duration (Sec), mean ± SD	Impedance drop (Ohm)	Slope of impedance course (Ohm/ms), median (IQR)	Power (W), mean ± SD	Temperature (°C), mean ± SD
**All points (n = 216)**	0.92 ± 0.83	0.42 ± 0.40	0.27 (3.72)	47.62 (92.40)	47.9 ± 10.5	5 (58)	–0.07 (1.19)	39.0 ± 4.3	36.1 ± 1.0
**Q1-weakest relative EGM voltage drop (n=50)**	0.66 ± 0.68	0.57 ± 0.57	0.05 (0.90)	11.95 (33.33)	46.6 ± 9.4	6 (47)	–0.07 (0.62)	39.5 ± 4.3	36.0 ± 1.0
**Q2-Intermediate weak relative EGM voltage drop (n = 48)**	0.72 ± 0.68	0.46 ± 0.46	0.18 (1.06)	36.04 (26.30)	48.8 ± 10.1	5 (57)	–0.06 (0.85)	39.1 ± 4.0	35.8 ± 1.0
**Q3-Intermediate strong relative EGM voltage drop (n = 67)**	0.82 ± 0.64	0.36 ± 0.27	0.30 (1.65)	54.17 (28.82)	47.6 ± 11.8	6 (55)	–0.08 (1.06)	38.9 ± 4.2	36.2 ± 1.1
**Q4-strongest relative EGM voltage drop (n = 51)**	1.51 ± 1.04	0.32 ± 0.23	0.95 (3.50)	76.00 (31.29)	48.6 ± 10.3	4 (48)	–0.08 (0.84)	38.7 ± 5.0	36.1 ± 1.0

**EGM voltage drops and the association with RF ablation CTI location**									
			**TV annulus-section**		**Mid-section**		**VCI-section**		

**All points** (n = 208)			58 (28%)		86 (41%)		64 (31%)		
**Q1-weakest relative EGM voltage drop** (n = 48)			15 (31%)		21 (44%)		12 (25%)		
**Q2-Intermediate weak relative EGM voltage drop** (n = 46)			9 (20%)		20 (43%)		17 (37%)		
**Q3-Intermediate strong relative EGM voltage drop** (n = 65)			19 (29%)		27 (42%)		19 (29%)		
**Q4-strongest relative EGM voltage drop** (n = 49)			15 (31%)		18 (37%)		16 (33%)		

Data are presented as mean ± SD, or median (IQR). CTI = cavotricuspid isthmus; EGM = electrogram; RF = radiofrequency; TV = tricuspid valve; VCI = vena cava inferior.

The results of the univariable analysis identifying determinants of relative EGM voltage drops are summarized in Table 4. Baseline EGM voltage showed the strongest association with relative EGM voltage drop (*P* < .001). From all patient characteristics evaluated, left ventricular ejection fraction (LVEF) and right atrium volume index (RAVI) showed a significant association with relative EGM voltage drop (*P* = .020 and *P* = .027, respectively).

**Table 4:** Predictors of relative EGM voltage drop during univariate analysis

Patient characteristics	B (95% CI)	*P* value
Sex	7.485 (–0.347, 15.317)	.061
Age	–0.117 (–0.528, 0.294)	.575
BMI	–0.110 (–1.110, 0.890)	.829
LVEF	–6.079 (–11.203, -0.955)	.020
LAVI	0.289 (–0.068, 0.646)	.112
RAVI	0.386 (0.045, 0.727)	.027
*RF ablation settings*		
Baseline EGM voltage	11.096 (7.503, 14.688)	<.001
Duration	0.142 (–0.167, 0.450)	.366
Impedance slope	–4.560 (–33.668, 24.547)	.758
Impedance fall	0.077 (–0.238, 0.393)	.630
Power	–0.398 (–1.139, 0.343)	.291
Temperature	0.725 (–2.400, 3.851)	.648
*CTI characteristics*		
CTI length iSuite TRA	0.383 (–0.696, 1.461)	.485
CTI length iSuite RAO	0.514 (–0.423, 1.451)	.281
CTI length MRI syst	–1.329 (2.147, –0.510)	.002
CTI length MRI diast	–0.693 (–1.437, 0.051)	.068
MRI CTI thickness	2.464 (–0.644, 5.573)	.120
Location CTI line	0.698 (–3.639, 5.035)	.751

BMI = body mass index; CTI = cavotricuspid isthmus; EGM = electrogram; LAVI = left atrial volume index; LVEF = left ventricular ejection fraction; MRI = magnetic resonance imaging; RAO = right anterior oblique; RAVI = right atrial volume index; TRA = transversal.

Among the anatomical parameters considered, systolic length, diastolic length and CTI thickness were associated with the relative EGM voltage drop (*P* = .002, *P* = .068, and *P* = .120, respectively).

RF ablation settings such as power and duration were moderately associated with the relative EGM voltage drop (*P* = .291 and *P* = .366, respectively).

### Clinical AFL recurrence and detailed characterization of late electrical CTI reconnection sites

Four of 12 patients (33%) underwent a conventional EP study within the 1-year follow-up: 3 patients for treatment of recurrent AFL and 1 patient for treatment of persistent atrial fibrillation. A high-density EAM and propagation map of the CTI line was performed in all patients during the conventional re-do EP study. In 1 patient with recurrent AFL, the electrical reconnection along the CTI was identified toward the TV annulus (Supplemental data Video 1). In the second patient with recurrent AFL, the electrical reconnection along the CTI was identified toward the VCI (Supplement data Video 2). In both cases, focal RF application at the site of electrical reconnection resulted in complete CTI block. In the third patient with recurrent AFL, no clear focal reconnection, no low-voltage areas, and no conduction delay could be identified in the high-density voltage and activation map, and a new linear RF ablation line was placed along the CTI, which resulted in complete CTI block. In the 1 patient with recurrent atrial fibrillation, the CTI line was complete.

The identified location of the electrical reconnection derived from the EAMs during the conventional re-do procedures were compared with the color-coded EGM voltage drop quartiles on the iSuite system and the post-ablation imaging of the index CMR-guided procedure, which included T2WI (n = 4), T_1_ mapping (n = 2), first-pass perfusion imaging during contrast administration (n = 2), and 2D dark-blood LGE (n = 2) sequences.

**Figure 3:**
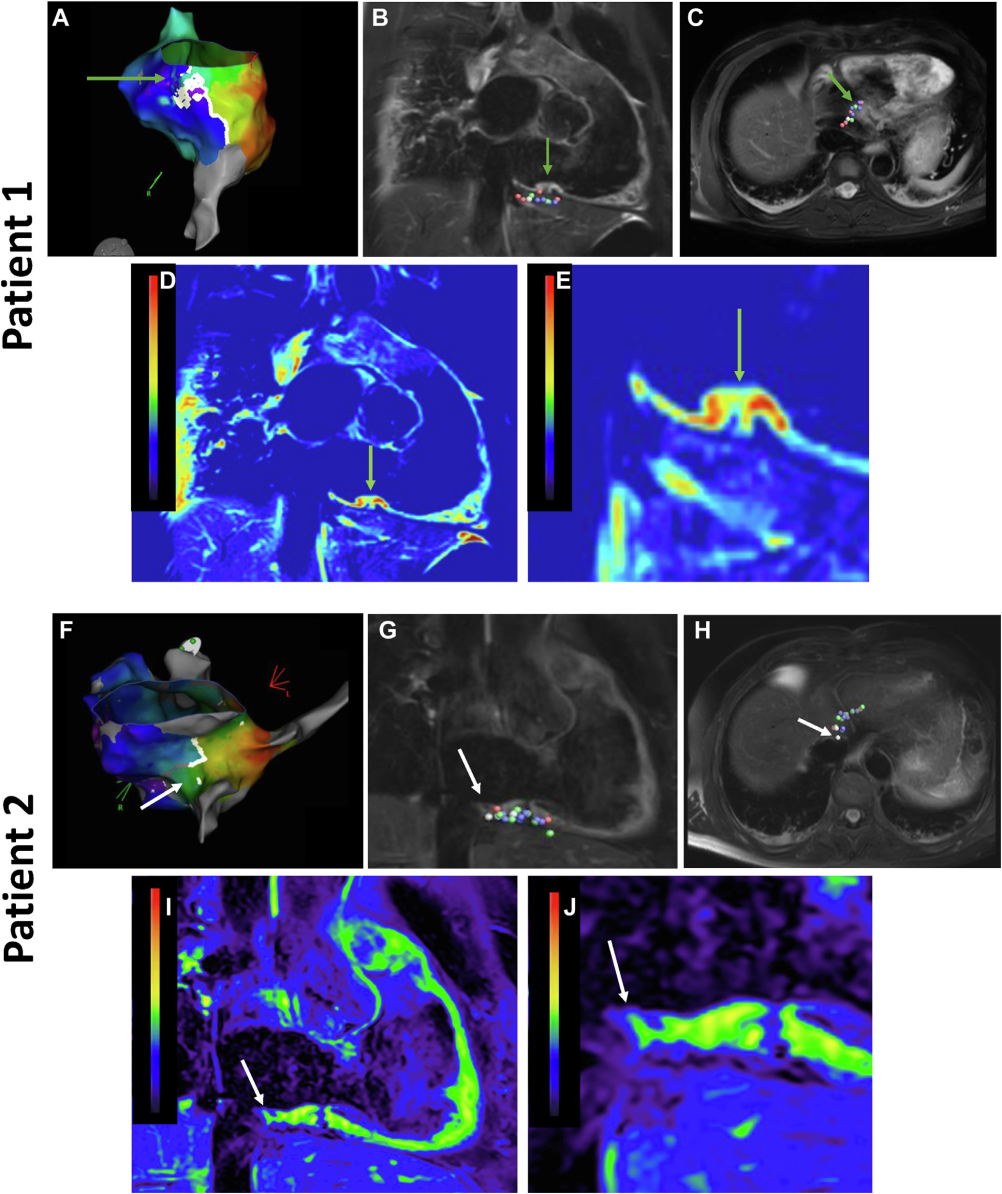
Patient 1 with recurrent atrial flutter (reconnection at tricuspid annulus). **A:** 3D electroanatomic voltage mapping of the RA during pacing from the coronary sinus shows an activation breakthrough towards the TV annulus in a LAO caudal view (green arrow). **B-E:** Post-ablation CMR images of the CTI line of the index procedure: color-coded distribution of the ablation lesions over projected on post-ablation T_2_-weighted CMR image in **B:** RAO and **C:** transversal view [green arrow points out the region with relatively low SI and the location of an RF application with intermittent weak EGM voltage drop (green point)] and **D:** T_2_-weighted heat map in RAO view where red and green colors represent areas with relatively higher and lower signal intensity when compared within this single image. **E:** Focus on the ablation line. Patient 2 with recurrent atrial flutter (reconnection at VCI). **F:** 3D electroanatomic voltage mapping of the RA during pacing from the coronary sinus shows an activation breakthrough toward the VCI (white arrow). **G–J:** Post-ablation CMR images of the CTI line of the index procedure: color-coded distribution of the ablation lesions over projected on post-ablation T_2_-weighted image in **G:** RAO and **H:** transverse view [white arrow points out the region with relatively low SI and the location of a RF application with weak EGM voltage drop (white point)] and **I:** T_2_-weighted heat map in RAO view where yellow and green colors represent areas with high and low SI, respectively. **J**: Focus on the ablation line. CMR = cardiac magnetic resonance; CTI = cavotricuspid isthmus; EGM = electrogram; LAO = left anterior oblique; RA = right atrium; RAO = right anterior oblique; RF = radiofrequency; SI = signal intensity; TV = tricuspid annulus; VCI = vena cava inferior.

In the 2 patients with late electrical reconnection along the CTI line, electrical gap overlapped with the location of RF applications with a weak EGM voltage drop (quartiles 1 and 2) and less signal intensity in the visual assessment of the qualitative T2WI during the index procedure (Figure 3). Although first-pass perfusion defects and LGE were present at the location of the ablation line, no regional differences could be visually identified. No T_1_ mapping was performed in those 2 patients.

## Discussion

This is the first detailed report on relative EGM voltage drops during CMR-guided CTI RF catheter ablation for the treatment of typical AFL. The herein established associations of relative EGM voltage drop with delivered RF energy, patient characteristics, CTI anatomy, and acute post-ablation lesion characteristics derived from periprocedural CMR imaging are novel and may have potential to be integrated into CMR-guided catheter ablation.

CMR-guided catheter ablation has previously proved to be feasible and safe. Various strategies for real-time catheter localization have been developed for CMR-guided catheter ablation procedures to support manual catheter maneuvering to the target location by real-time active catheter tracking and allow manual allocation of the ablation points. However, none of these softwares currently incorporate feedback on tissue contact and effectiveness of ablation lesion formation. This is one of the reasons why CMR-guided ablation is still limited to anatomical ablation procedures, such as CTI ablation for typical AFL. In these anatomical ablation procedures, catheter movements and tissue contact also can be confirmed by conventional interpretation of local bipolar EGM and RF energy, which is delivered to achieve an EGM voltage drop. Relative bipolar EGM voltage drop has been previously described as a parameter correlating with transmural lesions in the human atrium.^18^

In our analysis, we showed that local bipolar EGM voltage amplitude at the start of the ablation point was the strongest predictor for the relative EGM voltage drop. In the case of the CTI region, the sharpness and amplitude of bipolar EGMs is dependent on tissue contact, tissue properties (healthy vs remodeled atrium), and the direction of activation.^19^ We assessed the stability and correct positioning of the catheter tip mainly by the assessment of the local bipolar EGM. Large and stable EGM amplitudes are likely representing positions with stable and high tissue contact, which will result in a clear and reproducible EGM voltage drop during RF energy delivery.

Anatomical parameters showed an association with relative EGM voltage drops: right atrial dimension, the length of the CTI line measured during periprocedural MR imaging, and to a smaller extent the CTI thickness. Interestingly, the length of the ablation line along the CTI, which was assessed in the dedicated MRI navigation system (iSuite), was significantly shorter than the anatomical CTI lengths measured on MRI during systole and significantly longer than the anatomical CTI lengths measured on MRI during diastole and not related to the relative EGM voltage drop. The differences between the CTI length during systole and diastole represents the dynamic of the ablation target area attributable to the movement of the CTI line during the cardiac cycle. Because the ablation line was mainly guided by the presence of atrial EGMs, the difference between the length of the ablation line and the anatomically determined CTI length in systole indicates that some parts of the anatomical CTI do not have any atrial electrical signals and do not require RF ablation to achieve complete electrical block along the CTI. Another explanation may be the fact that during 1-minute RF application, the CTI line may constantly move below the catheter tip throughout the cardiac cycle, which may result in a larger ablation area than expected. Unfortunately, real-time active tracking visualizing catheter throughout the cardiac cycle results in strong interferences of the magnetic field with the EP system, inducing noise in the local EGMs, and therefore, we could not monitor the CTI and catheter movement during RF application. However, no specific anatomical location along the CTI line could be identified in which ablation points with particularly weak or strong EGM voltage drops occurred more frequently.

Surprisingly, duration and power of the RF energy delivery were related to a small extent with the relative EGM voltage drops during CMR-guided CTI ablation. The observation that RF energy delivery settings are related to EGM voltage and impedance drop is in agreement with previous studies with contact force sensing catheters.^20^ The amount of RF energy delivered from the CMR-compatible irrigated ablation catheter in an iCMR environment was higher (39 ± 4 W) than usually used for CTI RF ablations. Recent studies have shown that high-power (40–50 W) with irrigated ablation catheter tip during CTI ablation in a conventional EP laboratory has a lower first-pass success rate and higher probability for steam-pops, which were not observed during our study.^21^^,^^22^ Additionally, unstable and low-contact catheter positions, which cannot be assessed and controlled during CMR-guided ablation procedures, and interaction with the electromagnetic field of the MRI scanner itself may contribute to these findings.

In 2 patients with recurrent clinical AFL, EAM remapping data of the ablation line could be collected. In these 2 patients, the location of late electrical CTI reconnection sites during a conventional re-do procedure was characterized by ablation points in the individual weakest quartile according to EGM voltage drop during the index procedure. Additionally, the location of late electrical CTI reconnection sites in both patients was characterized by lower signal intensity during T2WI. Previous studies have shown that signal intensity in T2WI may be useful for a qualitative assessment of the presence of edema along the CTI ablation line because of the inflammatory changes caused by RF ablation.^23^^,^^24^ In the qualitative T2WI heat maps, regions of relatively low signal intensity can be identified easily by the treating electrophysiologists, who are used to visual interpretation of color-coded maps, which may therefore hold some promise for future real-time integration in CMR-guided catheter ablation procedures. However, although the CTI line could be anatomically localized during the first-pass perfusion imaging and LGE, visual interpretation failed to identify regional differences that could suggest later electrical gaps. This is in line with earlier preclinical and clinical studies that have shown that LGE exhibited additional enhancement of adjacent edematous tissues, causing misinterpretation of the RF lesion size.^25^^,^^26^ Unfortunately, EAM mapping data of the ablation line could not be systematically collected in all patients, which does not allow the sensitivity and specificity of post-ablation T2-weighted imaging to detect areas of late reconnection. Therefore, whether this correlation of EGM and T2WI, a sequence that may lack some accuracy because of limited spatial resolution and movement (breathing and respiration) artefacts, can be integrated into the CMR-guided catheter ablation procedures and improve outcomes warrants further studies with more advanced integrated EP systems and systematic remapping studies.

## Limitation

Our study population is rather small; however, the number of RF applications included is quite high, and because this is the first study to investigate the association of local EGM during CMR-guided CTI catheter ablation, no sample power was possible. There is no real-time integration of the periprocedural CMR imaging to the MRI-dedicated mapping software available; all analyses were made retrospectively, and that may bear some risk of interpretation. Whether a more advanced integration of EGM and periprocedural CMR imaging impacts CMR-guided ablation procedure times and ablation outcomes needs to be investigated in future studies.

## Conclusion

This is the first study to show that during CMR-guided catheter ablation of typical AFL the local atrial bipolar EGM voltage drop, as an indicator for durable ablation lesion, was mainly associated with the EGM voltage at the baseline, LVEF, RAVI, and the CTI length at ventricular end-systole. RF delivery settings and lesion parameters were less strongly correlated. In patients with a clinical recurrence of AFL, the qualitative T2WI during the periprocedural CMR imaging showed visually less signal intensity at the area of late reconnection.

## Disclosures

All authors have no conflicts to disclose.
