# Supplementary material for: Local atrial bipolar electrogram voltage drops during cardiac magnetic resonance guided catheter ablation of typical atrial flutter: Associations with delivered radiofrequency energy and peri-procedural imaging
Source: Heart Rhythm O2. 2024 Sep 6;5(11):778–87. doi: 10.1016/j.hroo.2024.08.015 (PMC11624345; doi:10.1016/j.hroo.2024.08.015)
Supplement: Supplementary Material [file mmc1.docx]

**Supplement data**

**Videos 1 and 2**

**Video 1** A 3D electroanatomic, voltage, and propagation map of the right atrium during pacing from the coronary sinus shows an activation breakthrough on the cavotricuspid isthmus line toward the tricuspid annulus in a left anterior oblique caudal view.

**Video 2** A 3D electroanatomic, voltage, and propagation map of the right atrium during pacing from the coronary sinus shows an activation breakthrough on the cavotricuspid isthmus line toward the vena cava inferior in a left anterior oblique caudal view.

**Supplemental Table 1** Typical CMR acquisition parameters

| **Protocol** | **Sequence** | **TE (ms)** | **TR (ms)** | **FA (^o^)** | **Slice thickness (mm)** | **In-plane resolution reconstructed (mm^2^)** | **Acceleration factor** |
| --- | --- | --- | --- | --- | --- | --- | --- |
| Cine imaging | bSSFP | 1.46 | 2.9 | 60 | 8 | 0.91 × 0.91 | 2.7  C-SENSE |
| T_2_WI | Black-blood turbo spin-echo SPIR | 70 | 2 × RR interval | n/a | 8 | 0.81 × 0.81 | 2.3  C-SENSE |
| T_1_ mapping | 5(3)3 MOLLI | 0.84 | 1.93 | 35 | 10 | 1.17 × 1.17 | 2.0  SENSE |
| First-pass perfusion | Saturation-recovery bSSFP | 1.0 | 2.2 | 50 | 10 | 1.46 × 1.46 | 2.0  SENSE |
| LGE | Dark-blood PSIR  spoiled-TFE | 3.0 | 6.1 | 25 | 8 | 0.8 × 0.8 | 1.8  C-SENSE |
| bSSFP = balanced steady-state free precession; (C-)SENSE = (compressed) sensitivity-encoding; FA = flip angle; LGE = late gadolinium enhancement; MOLLI = modified Look-Locker inversion-recovery; PSIR = phase-sensitive inversion-recovery; SPIR = spectral presaturation with inversion recovery; T_2_WI = T_2_-weighted imaging; TE = echo time; TR = repetition time. | | | | | | | |
